# COVID-19 Adaptations in the Care of Patients with Opioid Use Disorder: a Survey of California Primary Care Clinics

**DOI:** 10.1007/s11606-020-06436-3

**Published:** 2021-01-28

**Authors:** Lauren Caton, Hannah Cheng, Hélène Chokron Garneau, Tammy Fisher, Briana Harris-Mills, Brian Hurley, Sandra Newman, Mark P. McGovern

**Affiliations:** 1 Center for Behavioral Health Services and Implementation Research, Division of Public Mental Health and Population Sciences, Department of Psychiatry & Behavioral Sciences, Stanford University School of Medicine, Palo Alto, CA USA; 2 Center for Care Innovations, Oakland, CA USA; 3 Department of Family Medicine, University of California Los Angeles, Los Angeles, CA USA; 4 Los Angeles County Department of Health Services, Los Angeles, CA USA; 5 Division of Primary Care and Population Health, Department of Medicine, Stanford University School of Medicine, Stanford, CA USA

**Keywords:** COVID-19, opioid use disorder (OUD), medications for opioid use disorder (MOUD), telehealth, office-based opioid treatment (OBOT)

## Abstract

**Background:**

With the onset of the COVID-19 crisis, many federal agencies relaxed policies regulating opioid use disorder treatment. The impact of these changes has been minimally documented. The abrupt nature of these shifts provides a naturalistic opportunity to examine adaptations for opioid use disorder treatment in primary care.

**Objective:**

To examine change in medical and behavioral health appointment frequency, visit type, and management of patients with opioid use disorder in response to COVID-19.

**Design:**

A 14-item survey queried primary care practices that were enrolled in a medications for opioid use disorder statewide expansion project. Survey content focused on changes in service delivery because of COVID-19. The survey was open for 18 days.

**Participants:**

We surveyed 338 clinicians from 57 primary care clinics located in California, including federally qualified health centers and look-alikes. A representative from all 57 clinics (100%) and 118 staff (34.8% of all staff clinicians) participated in the survey.

**Main Measures:**

The survey consisted of seven dimensions of practice: medical visits, behavioral health visits, medication management, urine drug screenings, workflow, perceived patient demand, and staff experience.

**Key Results:**

A total of 52 of 57 (91.2%) primary care clinics reported practice adaptations in response to COVID-19 regulatory changes. Many clinics indicated that both medical (40.4%) and behavioral health visits (53.8%) were now exclusively virtual. Two-thirds (65.4%) of clinics reported increased duration of buprenorphine prescriptions and reduced urine drug screenings (67.3%). The majority (56.1%) of clinics experienced an increase in patient demand for behavioral health services. Over half (56.2%) of clinics described having an easier or unchanged experience retaining patients in care.

**Conclusions:**

Many adaptations in the primary care approach to patients with opioid use disorder may be temporary reactions to COVID-19. Further evaluation of the impact of these adaptations on patient outcomes is needed to determine whether changes should be maintained post-COVID-19.

Patient access to medications for opioid use disorder (OUD)—including buprenorphine and methadone—remains one of the largest hurdles to opioid use disorder treatment. Despite robust evidence of these medications’ impact on reducing mortality and overdose, fewer than one-third of patients receive them in any care setting.^1, 2^ Many barriers limit access: prescriber waiver requirements, concerns about medication diversion and misuse, stigma toward and internalized by patients, transportation availability, and mandatory in-person induction visits.^3–5^ Given the effectiveness and safety of these medications for opioid use disorder treatment,^1, 2^ there has been a long-standing call to ease access and remove barriers.^6–9^

The COVID-19 pandemic has caused major disruptions to the care of patients with opioid use disorder. California was among the first states with confirmed COVID-19 cases—with a daily average of 1678.4 new cases during the study period—and the first to employ a statewide shelter-in-place order.^10, 11^ State edicts prompted health care to move virtual and subsequent federal guidelines relaxed care restrictions. Though these changes present challenges for clinics, they also provide a naturalistic experiment on the removal of some barriers to OUD care delivery. Many treatment programs require in-person medication initiations, frequent visits, and negative urine drug screenings (UDS) before prescribing. These practices became challenging to maintain with the onset of COVID-19 restrictions.^12^ It is important to maintain continuity of care for patients with opioid use disorder, and the risk of adverse health outcomes—such as overdose and use recurrence—increases significantly with medication discontinuation.^13^ With the knowledge that patient retention is essential for opioid use disorder treatment, the Drug Enforcement Administration (DEA) dropped the in-person exam requirement for buprenorphine initiations, allowing prescribers to use telemedicine in late March.^14^ The Substance Abuse and Mental Health Services Administration (SAMHSA) also confirmed these changes.^15–17^ The Department of Health and Human Services (HHS) relaxed HIPAA rules, permitting health care providers—across medical specialties—to use non-public facing communications technology for telehealth appointments.^18^ With no federal mandate for urine drug screenings or frequent visits, clinics adjusted their own protocols to address this urgent need. Agencies only designated these reforms for the duration of the COVID-19 crisis, with an eventual return to precedent.

Addiction specialists are calling for the sustainable expansion of these new avenues for low-barrier care.^19–22^ It is therefore important to document clinic modifications resulting from relaxed federal guidelines. These unprecedented care delivery trends may provide lessons on reforms in the care of patients with opioid use disorder.

This report is the first to present primary care data on COVID-19 changes to the care of patients with opioid use disorder. We surveyed a sample of 57 primary care, predominantly federally qualified health care center (FQHC), practices. The resulting insights on staff experience with and practitioners’ preference for lower-barrier care may support re-evaluation of existing policies on the care of patients with opioid use disorder. Primary care practices may have the capacity and insight to lead the way.

## METHODS

### Participants

Participants included 338 staff members across 57 primary care clinics enrolled in an existing medication for opioid use disorder (MOUD) treatment expansion project. These primary care clinics include federally qualified health centers (FQHC), FQHC look-alikes, hospital-affiliated ambulatory care clinics, Indian Health Service, and rural health clinics. The clinics varied by number of employees, patient panel size, and stages of opioid use disorder care implementation (e.g., number of buprenorphine-waivered prescribers, number of patients prescribed buprenorphine). Clinic opioid use disorder care capability was determined in the previously mentioned study on opioid use disorder treatment expansion. Clinics were labeled *start-up* if they had a small number of x-waivered providers and few patients and *scale-up* if they were more established in their OUD care. These categorizations were developed by an expert team with qualifications in addiction medicine, addiction psychiatry, and primary care management and correspond to Aarons et al. Exploration, Preparation, Implementation and Sustainment (EPIS) stages: Exploration and Preparation (start-up OUD practice) and Implementation and Sustainment (scale-up OUD practice).^23^ Many clinics resided in rural and/or medically underserved areas. Study staff surveyed prescribers (medical director, physician, physician assistant, nurse practitioner), behavioral health personnel (social work, clinical psychologist, mental health therapist, substance use counselor, behavioral health manager), and others (nurse manager, nurses, medical assistant, clinical administrator, program coordinator).

### Survey Development and Measures

The 14-item survey collected data on how primary care clinics have adapted their practices for patients with opioid use disorder since the COVID-19 pandemic. Initial survey items came from DEA and SAMHSA pandemic-specific modifications to existing medication and opioid use disorder care guidelines. We sourced additional items from recommendations presented in a 3-hour-long webinar. The Center for Care Innovations hosted the webinars that were designed to provide pandemic transition support. Important practice change topics emerged as themes from the interactive discussion among attendees. Through an iterative process, a team of subject matter experts—including an addiction psychiatrist, a clinical psychologist, and two quality improvement specialists—further refined and finalized the survey.^24^

The final survey is composed of 14 items across seven domains: (i) medication visits, (ii) behavioral health visits, (iii) medication management, (iv) urine drug screenings, (v) workflow, (vi) patient demand, and (vii) staff experience. Each category assessed both frequency and delivery modalities. The survey had an open-ended textbox at the end of the survey for respondents to comment on their experiences with adaptations to their clinic practice. A sample domain with corresponding items is listed in Table 1.

**Table 1:** Sample Item from Participant Survey

Please indicate changes that have been made to **medication visit type** (in person = in clinics; virtual = phone or video) [Check all that apply]	
We have not made any changes to our in-person initial MOUD visits or follow-up MOUD visits	
We do not offer any virtual visits for patients starting MOUD	
Some visits for patients starting MOUD are in-person and some are virtual	
All visits for patients starting MOUD are virtual	
We do not do any virtual options for follow-up MOUD visits	
Some follow-up MOUD visits are in-person and some are virtual	
All follow-up MOUD visits are virtual	
In-person follow-up MOUD visits are less frequent but no longer than one month apart	
Follow-up MOUD visits are as frequent but a combination of in person and virtual	
Follow-up MOUD visits are as frequent and all virtual	
Follow-up MOUD visits are less frequent and all virtual	

#### Adaptation to Routine Medication Practices

To examine how clinics made adaptations to medication practice, respondents checked statements ranging from no adaptation (e.g., no changes) to complete adaptation (e.g., fully virtual telehealth for medications for opioid use disorder). Five practice adaptation categories were assessed: medical visits, behavioral health counseling visits, medication management, urine drug screenings (UDS), and clinic workflow. Respondents selected which statement reflected their current practice. Frequency (increase, decrease, no change) and modality (all virtual, all in-person, and combination) questions were asked for medication visits, behavioral health visits, urine drug screening questions (Table 1). Medication management and clinic workflow questions focused on duration, dose, and presence/absence of specified changes. The survey also requested a breakdown of percent of virtual visits, patients on buprenorphine injectable/implants, and patients on injectable naltrexone before and since April 2020. They could select multiple response options in each set of questions, marking all that apply.

#### Staff Perception of Patient Response and Staff Experience

For the sixth domain, respondents were asked to select items corresponding to their perception of COVID-19-related changes to patient care. These statements surveyed both patient demand for medication and counseling visits and staff perception of patient preference for delivery modality. The seventh domain evaluated staff experience during COVID-19. Respondents were asked to select wellness statements that apply to them. These statements describe staff experience with clinic staffing, work location, personal wellness, and clinic support.

### Procedure

The survey was administered between April 20 and May 8, 2020. An anonymous Qualtrics link was emailed to 338 staff members within the 57 primary care clinics. Program leadership sent three reminder emails, each spaced a week apart, to ensure timely response from staff members. The datasets analyzed during the current study are available from the corresponding author upon request.

### Ethics

Prior to survey distribution, a waiver of consent was obtained from the Stanford Institutional Review Board. An anonymous survey link was used to ensure the anonymity and confidentiality of participant data. No identifiable information was collected from participants.

### Statistical Analysis

Descriptive statistics depict respondent characteristics at the clinic and the staff level. To aggregate data at the clinic level, cases of discordant response (i.e., disagreement between the respondents from the same clinic) were resolved by selecting the response of the prescriber; if no prescriber response was present for a clinic, then the response of the leadership role (e.g., substance use director, nurse manager) was used. The study deferred to clinic leadership and prescriber responses given their clinical and administrative responsibilities. These roles likely had the most familiarity with the clinic’s opioid use disorder practice adaptations.

Aggregated response percentages assessed clinic-level and medication-specific adaptations since the pandemic. Chi-square tests were performed to examine differences in *start-up* versus *scale-up* clinic responses and in staff wellness comparison of leadership versus other staff members. All analyses were performed using SPSS Version 25 and R Version 4.0.1.

## RESULTS

At least one staff member from all 57 clinics completed the survey. Response rate for clinics was therefore 100% and that of individual staff members was 34.8% (118 out of 338 surveyed individuals). Respondents were evenly distributed across role category: prescribers (32.2%), behavioral health staff (27.1%), and others (33.9%). Prescribers include physicians, nurse practitioners, and physician assistants. The “Other” category is comprised of nurse manager, nurses, medical assistant, clinical administrator, and program coordinator. Fifty-one percent of clinics were *scale-up* in terms of opioid use disorder care capability. Forty-nine percent were *start-up* and in the early stages of integrating opioid use disorder care into their practice. Characteristics of participating clinics are presented in Table 2.

**Table 2:** Demographic Characteristics of Survey Respondents

**By clinic (*N*** **= 57)**		***N***	**%**
OUD care capability			
	Start-up	29	50.9
	Scale-up	28	49.1
Rurality and shortages			
	Urban/metropolitan	48	84.2
	Rural	9	15.8
	Medically underserved area (MUA)	23	40.4
Primary care clinics			
	FQHC	41	71.9
	FQHC look-alikes	2	3.5
	Hospital-affiliated ambulatory care clinic	8	14.0
	Indian Health Service	4	7.0
	Rural health clinics	2	3.5
Adaptations during COVID-19			
	Yes	52	91.2
	No	5	8.8
Organization patient panel size			
	Small (0–14,999 patients)	23	40.4
	Medium (15,000–59,999 patients)	17	29.8
	Large (≥ 60,000 patients)	17	29.8
		**Median**	**SD**
General clinic characteristics			
	Number of physicians	6.0	31.1
	Number of certified nurse practitioner	3.0	5.3
	Number of physician assistants	1.0	2.4
	Number of addiction-certified physicians	0.0	0.6
	Number of psychiatrists	0.0	1.0
	Number of addiction-certified psychiatrists	0.0	0.8
	Number of mental health– and addiction-certified behavioral clinicians	0.5	1.4
Opioid use disorder care clinic characteristics			
	Number of x-waivered prescribers	4.0	5.7
	Number of active x-waivered prescribers	3.0	3.5
	Number of patients with opioid use disorder	151.0	200.6
	Number of patients who prescribed medications	22.0	67.4
Insurance type			
	% patients on Medicaid	63.5	17.7
	% patients on Medicare	5.5	15.1
	% patients with dual eligibility	4.0	8.6
	% patients on private insurance	3.0	11.1
	% uninsured patients	15.0	13.3
**By individual (*N*** **= 118)**		***N***	**%**
Respondent role			
	Prescribers	38	32.2
	Behavioral health personnel	32	27.1
	Other	40	33.9
	Prefer not to answer	8	6.8
Leadership role	Clinic leaderships	24	20.3
	Other staff members	94	79.7

### Medication and Behavioral Health Visits

**Figure 1:**
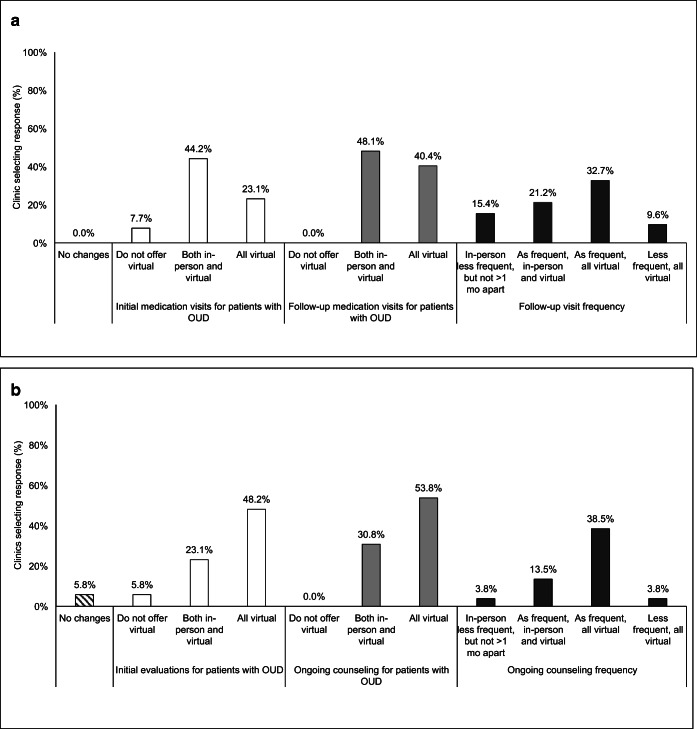
**Adaptations for initial and ongoing visits, by medication and behavioral/counseling services (*n*** **= 57). a Reported adaptations for medication visits. b Reported adaptations for behavioral health and/or counseling visits. Note: Clinics could select multiple response options; percentages may not add to 100%. All opioid use disorder (OUD) references are in acronym form.**

A majority (52/57, 91.2%) of clinics made adaptations to their opioid use disorder care practices in response to COVID-19. The majority of clinics reported that changes were made to both their medication and behavioral health visits (Fig. 1). Most notably, appointments transitioned to a virtual format. Remaining in-person visits tended to be for medication induction although some did occur virtually. Frequency of visits appears to remain unchanged.

We have increased telehealth visits for established and stable patients. However, it is too high-risk and liability is involved with new patients. We do not establish care or take new patients by telehealth or virtual visits. The risk does outweigh the reward in this case in our professional assessment and observation. We will provide follow up counseling, behavioral health, and medical appointments by phone for established and stabilized patients.—Substance use counselor

We had become more comfortable doing home inductions compared to before and having more success engaging them over the phone.—Medical Director

### Medication Management and Urine Drug Screenings

Clinics reported prescribing buprenorphine for longer durations (65.4%) than pre-COVID-19. Rates of injectable buprenorphine or naltrexone remained essentially unchanged pre- and post-COVID-19, with a slight 3.8% and 1.9% uptick, respectively. Clinics designated as *scale-up* in terms of opioid use disorder capabilities were significantly more likely to write prescriptions for longer durations than *start-up* clinics (*P* = 0.03). Sixty-seven percent of clinics reduced the frequency of urine drug screenings for established patients.

### Workflow Adaptations

The most significant workflow adjustments were as follows: changes in CPT codes to bill for virtual visits (59.6%), more assertive outreach to patients (48.1%), and lowered barriers for patients to start and continue on medications (61.5%).

### Patient Demand

**Figure 2:**
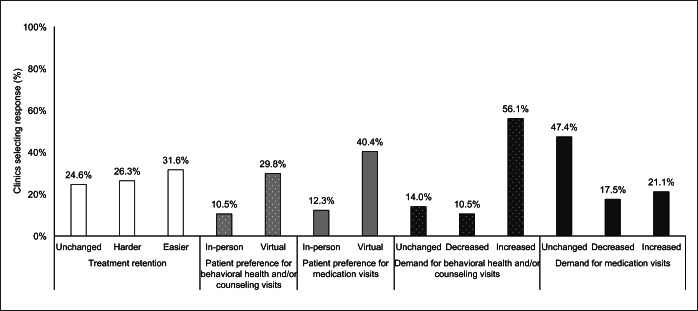
**Adaptations for patient retention, preference, and demand, by visit type (n = 57). Note: Clinics could select multiple response options; percentages may not add to 100%.**

Reporting of patient retention and engagement was about equally distributed across response options. Clinics reported having an easier (31.6%), harder (26.3%), and unchanged (24.6%) experience engaging patients and keeping them in care. Clinic reporting of patient experience during COVID-19 indicated an increase in demand for both medication and behavioral health visits, and perceived increase in patient preference for virtual visits (Fig. 2). This was also reflected in the qualitative responses:

We found out that we can get more patients to keep their appointments with telehealth.—Physician

Our patients prefer the in-person visits but have adapted to virtual/phone—Physician

Our group participation has gone way up to the point we are thinking of keeping it on Zoom.—MAT Program Manager

### Staffing Changes

Changes to clinic staffing, location, wellness, and support were reported at the individual level. About one-third of staff members (33.1%, 39/118) report some layoffs at their clinic while 23% report reduced hours but no layoffs. Seventy percent of staff members reported working partly onsite and partly at home with only 3.5% reporting working in the clinic as-usual. Some reported that their anxiety has impacted their functioning at home and work and that they are having a difficult time balancing home life and work. Most staff members, 64.9%, felt supported by their organization during this pandemic. However, the proportion of leadership staff reporting feelings of support is significantly higher, 79%, than that of non-leadership staff, 53% (*P* = 0.02). These changes were echoed in the qualitative responses by staff:

Collaboration between teams (behavioral health, primary care, MAT/OBOT team, whole person care team, etc) is much reduced.—Medical Director

I feel like I am working harder at home, being paid less, being pushed by management to increase productivity, and trying to balance home life with children who are largely being left to fend on their own during clinic hours.—Anonymous

## DISCUSSION

### Summary of Findings

Given the federal relaxation of practice guidelines in the care of patients with opioid use disorder during the COVID-19 era, changes to delivery were expected. This study documents the nature of these changes for a small subset of primary care practices. Almost all clinics, 91.2%, reported adaptations since April. Almost half of all medication and behavioral health visits moved to virtual formats with similar reported frequency. Demand for behavioral health and counseling services increased, while demand for medication-specific care remained largely unchanged and stable. Over half of practices report unchanged or easier experiences retaining and engaging patients in care. Practices reported prescribing longer doses of buprenorphine and reduced frequency of urine drug screenings.

Several of these changes were expected and may be explained, in part, by existing literature. Social supports and positive reinforcements that aid in recovery and prevent relapse may be absent for patients during “shelter-in-place” orders and social distancing requirements.^25^ As such, recent commentary in the field warned of an increased need for behavioral health support during COVID-19, reflected in these survey results.^20, 22^ Practitioners in our survey reported prescribing longer doses of their medications, significantly higher for clinics with higher OUD capabilities. This, along with decreases in urine drug screenings, may indicate renewed patient trust and prescribers prioritizing patient safety before their concerns for diversion and misuse.^3^ The divergence in reported support between leadership and non-leadership staff is striking and should be remedied to support achievement of the quadruple health care aim and address possible pandemic burnout.^26^ This aim emphasizes improvements to work-life of health care providers in additional to better health, better care, and lowest cost to patients.^27^ Even in the advent of significant protocol adaptations, approximately two-thirds of clinics report unchanged or easier time retaining and engaging patients. This is a key finding worth investigation as patient retention and care engagement are essential for reducing overdose and adverse health event risk.^13^

### Limitations and Next Steps

Inherent in survey data, these results are largely descriptive and non-causal. The small, and predominantly FQHC makeup of the sample, limits generalizability beyond primary care. Clinics in the sample were already prescribing medications and enrolled in an opioid use disorder expansion program. This could indicate a bias in acceptability and willingness to prescribe medications for the care of patients with opioid use disorder compared to other practices. Patient preferences were reported by clinicians, which is an inadequate proxy for accurate measure of patient experience. Only 38 prescribers (32.2%) responded across the 57 clinics and respondent role (prescribers, nurse practitioners, and administrators) varied—though evenly distribution—between clinics. Differential access to information about clinic functionality and perspectives between leadership, prescribers, and non-prescribers may limit possible comparison between clinics. Given the lack of temporal markers, it is difficult to assign motivations (e.g., a desire to social distance or an inclination for ease of access) to reported preferences for virtual care. Future studies should explore if patients themselves prefer virtual opioid use disorder and behavioral health care, with and without the desire and pressure to social distance. The field should also explore the impact of relaxed regulations during the COVID-19 pandemic on opioid treatment programs (OTPs). These clinics face steeper restrictions, and required in-person appointments for schedule-II medications prior to COVID-19. The long-term impact of these changes in relation to patient retention and outcomes is still unknown. The onset of the COVID-19 pandemic has provided a natural experiment on the removal of barriers to opioid use disorder care. Researchers should take advantage of these changes to advance our understanding of treatment access in the primary care and substance use treatment fields.

Both individuals with opioid use disorder and those at risk for COVID-19 share high rates of chronic health comorbidities, tenuous housing, and social risk factors.^19, 20, 22^ There is an immediate need to evaluate the intersection of these two crises. This study documents primary care opioid use disorder practice adaptations in response to COVID-19. It reveals a transition to virtual care for medications for opioid use disorder and significantly increased demand for behavioral health services. Telehealth may reduce transportation barriers and COVID-19 infection risks for patients and practitioners in the short term. Re-evaluating policies such as in-person appointments and medication dosage requirements may expand care access for patients with opioid use disorder in the long term. Calls to relax licensure requirements for prescribing, reduce prescribing requirements, and increase telemedicine predated the COVID-19 crisis.^21, 28^ Management of opioid use disorder should be treated like any other chronic health condition. The field should evaluate which protocols hinder or advance this outlook in order to tackle this complex illness.^29^ It is unclear whether changes to treatments for patients with opioid use disorder will or should perpetuate after the COVID-19 crisis.^21^ As many of the pandemic-precipitated adaptations are not exclusive to opioid use disorder management, this reckoning of re-evaluation should extend across all transformations of medical practice.

## References

[1] Volkow ND, Frieden TR, Hyde PS, Cha SS (2014). Medication-Assisted Therapies — Tackling the Opioid-Overdose Epidemic. New England Journal of Medicine..

[2] Nielsen S, Larance B, Degenhardt L, Gowing L, Kehler C, Lintzeris N. Opioid agonist treatment for pharmaceutical opioid dependent people. Cochrane Database of Systematic Reviews. 2016;2016(5). 10.1002/14651858.CD011117.pub2. 10.1002/14651858.CD011117.pub2 27157143

[3] Doernberg M, Krawczyk N, Agus D, Fingerhood M (2019). Demystifying buprenorphine misuse: Has fear of diversion gotten in the way of addressing the opioid crisis?. Substance Abuse..

[4] Huhn AS, Dunn KE (2017). Why aren’t physicians prescribing more buprenorphine?. Journal of Substance Abuse Treatment..

[5] Hutchinson E, Catlin M, Andrilla CHA, Baldwin LM, Rosenblatt RA (2014). Barriers to primary care physicians prescribing buprenorphine. Annals of Family Medicine..

[6] Samet JH, Botticelli M, Bharel M (2018). Methadone in primary care - one small step for congress, one giant leap for addiction treatment. New England Journal of Medicine..

[7] Haffajee RL, Bohnert ASB, Lagisetty PA (2018). Policy Pathways to Address Provider Workforce Barriers to Buprenorphine Treatment. American Journal of Preventive Medicine..

[8] Fiscella K, Wakeman SE, Beletsky L (2019). Buprenorphine Deregulation and Mainstreaming Treatment for Opioid Use Disorder: X the X Waiver. JAMA Psychiatry..

[9] Davis CS, Carr DH (2019). Legal and policy changes urgently needed to increase access to opioid agonist therapy in the United States. International Journal of Drug Policy..

[10] Johns Hopkins Coronavirus Resource Center. COVID-19 United States Cases by County. https://coronavirus.jhu.edu/us-map. Accessed 6 Oct 2020.

[11] Johns Hopkins Coronavirus Resource Center. Impact of Opening and Closing Decisions by States. https://coronavirus.jhu.edu/data/state-timeline. Accessed 6 Oct 2020.

[12] Dunlop A, Lokuge B, Masters D (2020). Challenges in maintaining treatment services for people who use drugs during the COVID-19 pandemic. Harm reduction journal..

[13] Williams AR, Samples H, Crystal S, Olfson M (2020). Acute Care, Prescription Opioid Use, and Overdose Following Discontinuation of Long-Term Buprenorphine Treatment for Opioid Use Disorder. Am J Psychiatry.

[14] Drug Enforcement Administration. COVID-19 Information Page. Diversion Control Division. https://www.deadiversion.usdoj.gov/coronavirus.html. Accessed 15 Jul 2020.

[15] Department of Justice, United States. Use of Telemedicine While Providing Medication Assisted Treatment (MAT).; 2018.

[16] Substance Abuse and Mental Health Services Administration. Opioid Treatment Program (OTP) Guidance. www.samhsa.gov. Accessed 15 Jul 2020.

[17] Substance Abuse and Mental Health Services Administration. FAQs: Provision of Methadone and Buprenorphine for the Treatment of Opioid Use Disorder in the COVID-19 Emergency. https://www.samhsa.gov/sites/default/files/otp-guidance-20200316.pdf. Accessed 15 Jul 2020.

[18] U.S. Department of Health & Human Services, Office for Civil Rights. Notification of Enforcement Discretion for Telehealth. Health Information Privacy. Published March 30, 2020. https://www.hhs.gov/hipaa/for-professionals/special-topics/emergency-preparedness/notification-enforcement-discretion-telehealth/index.html. Accessed 15 Jul 2020.

[19] Vecchio S, Ramella R, Drago A, Carraro D, Littlewood R, Somaini L. COVID19 pandemic and people with opioid use disorder: innovation to reduce risk. Psychiatry Research. 2020;289. 10.1016/j.psychres.2020.113047. 10.1016/j.psychres.2020.113047 PMC7190508 32387795

[20] Marsden J, Darke S, Hall W, et al. Mitigating and learning from the impact of COVID-19 infection on addictive disorders. Addiction. 2020;115(6). 10.1111/add.15080. 10.1111/add.15080 PMC9364227 32250482

[21] Green TC, Bratberg J, Finnell DS (2020). Opioid use disorder and the COVID 19 pandemic: A call to sustain regulatory easements and further expand access to treatment. Substance Abuse..

[22] Davis CS, Samuels EA. Opioid Policy Changes During the COVID-19 Pandemic - and Beyond. Journal of addiction medicine. Published online 2020. 10.1097/ADM.0000000000000679. 10.1097/ADM.0000000000000679 PMC7273953 32433363

[23] Aarons GA, Hurlburt M, Horwitz SM (2011). Advancing a conceptual model of evidence-based practice implementation in public service sectors. Adm Policy Ment Health.

[24] McGovern MP, Hurley B, Fisher T, Caton L. Primary Care Practice Adaptations for Patients with Opioid Use Disorder during COVID-19: A Survey. 10.1007/s11606-020-06436-3 PMC7842998 33511572

[25] Bickel WK, Johnson MW, Koffarnus MN, MacKillop J, Murphy JG (2014). The Behavioral Economics of Substance Use Disorders: Reinforcement Pathologies and Their Repair. Annual Review of Clinical Psychology..

[26] Oser CB, Biebel EP, Pullen E, Harp KL (2013). Causes, Consequences, and Prevention of Burnout among Substance Abuse Treatment Counselors: A Rural versus Urban Comparison. J Psychoactive Drugs.

[27] Bodenheimer T, Sinsky C (2014). From triple to Quadruple Aim: Care of the patient requires care of the provider. Annals of Family Medicine..

[28] Yang YT, Weintraub E, Haffajee RL (2018). Telemedicine’s Role in Addressing the Opioid Epidemic. Mayo Clin Proc.

[29] McLellan AT, Lewis DC, O’Brien CP, Kleber HD (2000). Drug Dependence, a Chronic Medical Illness: Implications for Treatment, Insurance, and Outcomes Evaluation. JAMA..

